# Children Receiving a Nutrition and High-Quality Early Childhood Education Intervention Are Associated with Greater Math and Fluid Intelligence Scores: The Guatemala City Municipal Nurseries

**DOI:** 10.3390/nu14071366

**Published:** 2022-03-25

**Authors:** Ana M. Palacios, Lisa M. Villanueva, Matthew B. Flynn, Erik Parker, Stephanie Dickinson, Helen W. Bland, Greg A. Reinhart

**Affiliations:** 1 Department of Health Policy and Community Health, Jiann Ping Hsu College of Public Health, Georgia Southern University, Savannah, GA 31419, USA; hwbland@georgiasouthern.edu; 2 The Mathile Institute for the Advancement of Human Nutrition, Guatemala City 01009, Guatemala; lisa@mathileinstitute.org; 3 Department of Political Sciences and International Studies, Georgia Southern University, Statesboro, GA 30460, USA; mflynn@georgiasouthern.edu; 4 Biostatistics Consulting Center, Indiana University Bloomington School of Public Health, Bloomington, IN 47405, USA; erikpark@indiana.edu (E.P.); sd3@indiana.edu (S.D.); 5 The Mathile Institute for the Advancement of Human Nutrition, Dayton, OH 45414, USA; reinhart.greg@gmail.com

**Keywords:** undernutrition, stunting, overweight, early education, micronutrient supplement, childcare, global nutrition, Guatemala, mathematics, fluid intelligence

## Abstract

Background: About 47% of children < 5 years of age are stunted in Guatemala. In this study, the investigators aimed to compare growth and cognitive outcomes between children in second grade that attended five Guatemala City Municipal Nurseries (GCMN) vs. same sex, grade, and age-matched children. Methods: A cross-sectional design nested in a retrospective cohort was implemented between 2015 and 2019. Children that attended the GCMN and matched controls completed a math test and validated receptive language and fluid intelligence tests. The primary caregivers completed a sociodemographic survey. General and generalized linear mixed effect models were used to compare children that attended the GCMN vs. controls. The models were adjusted by maternal education, sex, asset score, and other relevant covariates. Results: Children that attended the GCMN exhibited greater math and fluid intelligence scores relative to the controls in the adjusted models (ß = 6.48; 95% CI (2.35–10.61)) and (ß = 1.20; 95% CI (0.12–2.29)), respectively. Lower odds of stunting were significant for children who went to any early childcare institution (AOR = 0.28; 95% CI (0.09–0.89)). Conclusions: The importance of integrating nutrition and high-quality early childhood education interventions in cognitive and growth outcomes is highlighted in this study. The GCMN model may be a scalable model in similar low-resource settings.

## 1. Introduction

Early life factors, including health, nutrition, security and safety, responsive caregiving, and early learning opportunities, are key to development, especially during the first years of life, and likely have lasting effects in an individual’s life [1]. Worldwide, it is estimated that 39% of children under five years of age in low- and middle-income countries are at risk of not reaching their full developmental potential, leading to an estimated deficit of 19.8% in adult annual income [2]. Children that suffer from nutritional deficiencies during the first years of life have greater odds of suffering from chronic, non-communicable diseases during adulthood [3,4,5]. Stunting, defined as a length/height-for-age z-score < −2, is a common manifestation of persistent micronutrient deficiencies [6].

The nutritional status during early childhood is related to cognitive and schooling achievement later in life [7,8,9,10]. The extent to which stunted children can catch up and achieve population reference heights and cognitive outcomes, and whether this catch-up can reverse the damage from chronic undernutrition during the first years of life, is still unclear.

In Guatemala, 46.5% of children under 5 years of age have stunting [11], the highest prevalence reported in the Western Hemisphere [11,12]. Several efforts to address nutritional deficiencies have been adopted in Guatemala in the past fifteen years. In 2005, the Guatemalan government made food security a national right, and several strategies to improve nutrition countrywide were developed [13]. Unfortunately, little improvement in reducing stunting has been documented from the national maternal and child health surveys from 2008–2009 and 2014–2015, which only showed a reduction of three percentage points, from 50% to 47% [11,14]. The current initiatives to reduce stunting in Guatemala included the provision of fortified corn–soy blends, lipid-based nutrient supplements, and micronutrient powders with diverse formulations that range from 2.0 to 8.0 mg/portion of zinc and 2.8 to 8.0 mg/portion of iron [15]. These initiatives are usually integrated with Guatemala’s Ministry of Health, which recommends well child-visits and/or partnerships nonprofit organizations. However, reaching the most vulnerable children, specifically young children under 5 years of age, continues to be a challenge, especially in rural areas and low-income urban neighborhoods.

In the area of education, access to early childhood education centers and preschools in Guatemala is unknown but thought to be scarce. Many of these initiatives are private and, thus, limited to families from higher sociodemographic backgrounds. Additionally, anecdotal evidence from investigators’ observations in the field suggest that many of these initiatives are predominant in middle- to high-income urban areas. According to the National Institute of Statistics in Guatemala, the net rate of children (ages 5 to 6 years) enrolled in early childhood education (pre-primary) is only 46.8% in the whole country [16]. To our knowledge, there is no robust data available on the coverage of early education in younger ages in this country.

The quality of early education offered in such institutions in Guatemala is largely unknown. However, the importance of high-quality early education and proper nutrition in cognitive development is well-documented to make lasting differences in the developmental potential of children [1].

In an effort to support women in the workforce and provide a safe space for children with low resources in the Guatemalan capital, the municipality of Guatemala City opened initially five nurseries (now eight) that offer heavily subsidized, high-quality early childhood services, including age-appropriate early education, health, and nutrition, to low-sociodemographic households. Located in underserved neighborhoods, these Guatemala City Municipal Nurseries provide care for young children living in poverty from Monday to Friday between 7:00 a.m. and 4:00 p.m. in the capital city. These nurseries offer child protection and daily care, early and pre-school education, nutrition, health, and foster positive values in the children.

Since 2010, children have received an age and culturally appropriate early childhood curriculum in addition to a comprehensive nutrition intervention that consisted of providing 75% of their daily macronutrient requirements distributed over a mid-morning snack, lunch, and a mid-afternoon snack. The morning snack is complemented by a 21 micronutrient-fortified beverage containing high concentrations of bioavailable chelated iron and zinc (12 mg and 9 mg, respectively) daily, provided as a corn–soy beverage (Chispuditos^®^). Young children receiving this nutritional supplement in a similar context were associated with better nutritional and health outcomes [17]. In addition, a local nutritionist engages with the teachers, cooks, and school staff, providing information sessions about every six months on topics that include age-appropriate nutrition and the importance of some micronutrients in the children’s diets. The cooking staff also receives training in the preparation and serving of foods and supplements using community engagement methods.

The objective of this study was to compare growth outcomes, mathematics scores, fluid intelligence, and receptive vocabulary between children in second grade that attended the Guatemala City Municipal Nurseries (GCMN) vs. sex, grade, and age-matched children that did not attend the municipal preschools.

## 2. Materials and Methods

A cross-sectional design nested in a retrospective cohort was implemented between 2015 and 2019. The study was approved by the Ethics Committee of the Universidad Francisco Marroquin protocol number CE/FM-UFM 0035-14.

In Guatemala, academic school years begin in January and finish in October. In the months of February and March, the primary caregivers of children that attended the Guatemala City Municipal Nurseries (GCMN) for 2 or more years were contacted via phone by a social worker who explained the nature of the study. If the mother showed interest in participating, a verbal prescreening survey was conducted. Eligible caregivers were then invited to a study session on a prespecified date. For the control group (children who did not attend the GCMN), researchers contacted the primary schools from the children that attended the GCMN in an effort to match the schools as much as possible. The study researchers shared the study objectives and procedures with the school directors and parents during a parent–teacher meeting. Those families that showed interest in participating were prescreened verbally. If children met the eligibility criteria, they were invited to the same study session.

Eligibility: Children living in Guatemala City and enrolled in second grade with no known language or learning disability were invited to participate. Inclusion for children that attended the GCMN consisted of having attended one of the five oldest municipal nurseries for two or more years. The children who were recruited as the “reference or control group” must have been attending the same elementary school or a comparable school to the GNC children and were individually matched based on sex, grade, and age range within 12 months.

**Figure 1:**
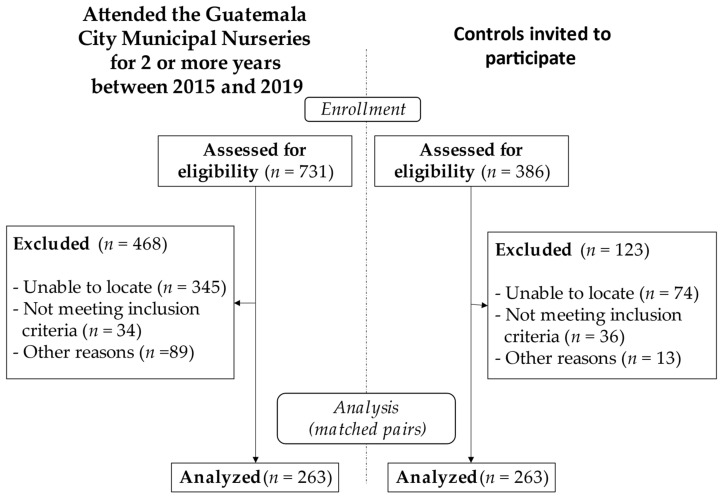
Consort diagram.

The present analysis included 263 sex- and aged-matched case–control pairs whose age differences were less than 12 months, with no diagnosed learning disability or language/speech delay (Figure 1, consort diagram).

During the study session, a consent form was read out load, and a copy was shared with each family. Signatures were collected from primary caregivers if they agreed to participate in the study. Participants then proceeded to complete the study activities.

Both children that attended the Guatemala City Nurseries and those in the “control group” were invited to attend the study data collection locations on a specific date, where primary caregivers were asked to complete a sociodemographic, nutrition, and health survey. Anthropometry was collected by two trained nutritionists following a detailed protocol [18]. Weight was measured using the digital weight scale Tanita, HD 314^®^ (Tanita, Chicago, IL, USA) to the nearest 0.1 kg. Standing height was measured using Seca 217^®^ stadiometers (Seca, Birmingham, UK) to the nearest 1 mm. Child’s anthropometric z-scores were calculated using the WHO 2007 Child Growth Reference [19]. The anthropometric measurements at the time were measured by the same trained nutritionists who measured the children for the present study. Stunting was defined as height-for-age z-scores (zHAZ) <−2. Underweight was defined as weight-for-age z-scores (zWAZ) <−2. Overweight/obesity was defined as BMI-for-age z-scores (zBMI) >+2. Emaciation was defined as weight-for-height z-scores (WHZ) <−2.

To determine household economic resources, 14 household items from the 2014 Guatemala National Survey of Living Conditions were included in the questionnaire [20]. An assets variable was created by adding all positive answers, with higher scores indicating more assets. The household income was self-reported and divided into quartile categories. Overcrowding was defined as more than three people per habitable room [21].

Maternal education was defined as having achieved secondary or more education or primary or less. Academic performance for the child was calculated averaging the individual numeric grades of the following main academic areas at the end of second grade: mathematics, science, social studies, and language.

Three psychologists who were trained for the study implemented a battery of validated tests that included the following: Raven’s progressive matrices, which measure fluid intelligence [22], the Peabody Vocabulary test that measures receptive vocabulary, [23], and a standard age-appropriate mathematics test (EVAMAT) for children in second grade that measures the children’s performances in the areas of arithmetic, calculus, geometry, and problem-solving abilities and had already been used in the region [24].

Data were entered onto a Microsoft Excel^®^ database (Microsoft, Redmond, WA, USA), and the database was exported to R v4.0.3 [25] and RStudio v1.2.1335 [26] using the packages car [27], and lme4 [28] for analysis.

Univariate analyses (χ^2^ for binary and two-tailed *t*-tests for continuous variables) were performed to compare the sociodemographic variables among children that attended the GCMN and reference children. The results were considered significant when the *p*-value was ≤0.05.

To compare children that attended the Guatemala City Nurseries vs. age, sex, and grade-matched controls that did not attend the nurseries, cross-sectional general and generalized linear mixed effects models were constructed. These models included random effects for each data collection year, primary school, and case/control pair group. Fixed-effect significance was assessed using Satterthwaite adjusted F-tests or Wald χ-square tests, and standard regression diagnostics were performed on each model.

Comparisons between “graduates” and “controls” were performed using linear mixed models for continuous variables, and logistic generalized linear mixed models for binary variables. All models were adjusted by sex, child’s age in months, maternal education, household asset score, and attendance in an early education or preschool institution, as some controls did not attend any preschool or preparatory education. Models predicting anthropometry outcomes were further adjusted by maternal BMI for the following outcomes: BMI-for-age z-score and overweightness. For the height-for-age z-score and stunting, outcome models were further adjusted by maternal short stature.

To avoid an inflation of type I error rates in the results from this study, the school clusters, data collection year, and case/control pair group were all included in the models as random effects [29].

## 3. Results

No differences between the GCMN graduates and controls were observed in age, *p* = 0.139, maternal education, *p* = 0.065, household asset score, *p* = 0.09, household income, *p* = 0.281, and living in an overcrowded home *p* = 0.671 (Table 1). While all CGMN graduates attended preschool, 8.7% of the controls did not attend any preschool or preparatory education.

### Anthropometry

Linear growth and stunting: In adjusted models, no significant differences between the GCMN graduates and controls (Table 2) were observed. However, the odds of stunting were 97% lower for any children who attended any early childcare institution, relative to those who did not attend one (AOR = 0.03; 95% CI (0.01–0.58); *p* = 0.02).

BMI and overweight/obesity: No differences were observed in the BMI-for-age z-scores (zBMI), underweight or overweight/obesity in both groups.

Academic and cognitive performance: Table 3 shows the mathematic, fluid intelligence, and receptive vocabulary test and academic average linear mixed models.

Mathematic scores: Children from the GCMN, relative to the controls, were more likely to have higher scores in mathematics (ß =6.48, *p* = 0.002), specifically in the areas of calculation (ß = 2.20, *p* = 0.002) and geometry (ß = 1.80, *p* = 0.011).

Fluid Intelligence: Children from the GCMN were associated with significantly higher fluid intelligence scores in both adjusted (ß =1.20, *p* = 0.029) and unadjusted (ß = 1.42, *p* = 0.011) models relative to the controls.

Receptive vocabulary and general average: No associations were observed between cases or controls for receptive vocabulary and general average in the adjusted models.

## 4. Discussion

In this study, researchers analyzed data from an urban sample of 8-year-old children attending second grade in Guatemala City. Growth outcomes, math performance, receptive language, and fluid intelligence were compared vs. sex-, grade-, and age-matched children. The findings from this study associate having attended the GCMNs with greater math and fluid intelligence scores relative to the controls, and children that ever attended an early childcare institution prior to elementary school had 72% lower odds of being stunted, relative to children who did not attend any.

The first few years of a person’s life are decisive to achieve a person’s maximum developmental potential. Adversities experienced during this period shape the functional and structural aspects of the psychology and biology of individuals and affect their immediate and future health, productivity, and wellbeing [30]. Stressors such as poverty, undernutrition, stress, and violence, among others, can negatively influence normal development and health, which may persist throughout the lifespan and into future generations [31,32].

In this study, we found that having attended the GCMNs was associated with higher scores in 2 of 4 areas of mathematics, including arithmetic, geometry, and calculation, and greater scores in fluid intelligence, relative to the controls, after adjusting for covariates. The development of early mathematical skills and fluid intelligence during preschool years may be an important factor associated with later success in math, reading, and academic attainment [33,34,35,36]. Children with weak math skills in the first school years are more likely to remain behind their peers in later years and are more prone to school desertion [37,38]. Adequate nutrition and early learning opportunities at home, in preschools, and in other social settings interact with each individual’s inherent ability to acquire skills, knowledge, and to solve problems: this capacity is referred as fluid intelligence [39]. Researchers from multiple studies have documented the significant improvement of fluid intelligence after improved nutrition and micronutrient supplementation [40,41]. Based on the findings of the present study, we suggest the conclusion that the quality of the services offered by the GCMN create better child outcomes than other alternatives in the area.

A major finding of this study was a significantly lower odd of stunting in children that attended any type of early childcare center prior to starting elementary school. Childcare centers as a platform to improve the nutritional outcomes are an emerging area in the literature. Black et al. (2021) evaluated the effectiveness of point-of-use micronutrient powder, relative to placebo fortification, in child development, growth, and morbidity in a 22-rural preschool-based randomized controlled trial [42]. The prevalence of anemia and iron deficiency was significantly reduced in children receiving the micronutrient powder. Further, effects of micronutrient supplementation in low-quality childcares influenced child development, as children from low-quality preschools showed significantly greater scores in expressive languages, inhibitory control, and social–emotional development, relative to the placebo [42]. Other researchers have also observed findings specific to growth outcomes. In Guatemala, Ruel and collaborators (2006) observed that children attending the “Hogares Comunitarios” (community-based daycare programs) consumed 20% more energy, protein, and iron and 50% more vitamin A than children that were not part of the program [43]. In Colombia, significant baseline-to-follow-up improvements in the prevalence of being underweight and wasting were observed in children ages 25–48 months after attending community-based daycares between 5 and <16 months [44].

More importantly, in a country where about one in every two children under five years have chronic undernutrition, quality childcare centers offer a tremendous opportunity to effectively ameliorate this problem. Interventions to improve the nutrition, health, and wellbeing of children worldwide have focused on the first 1000 days (from conception up to 24 months). However, the preschool years are characterized by continued brain development and developmental plasticity [45]. The findings from this study offer further support that providing comprehensive, scalable services that include a safe environment, rich in early learning opportunities, and nutrition interventions with macro-and micronutrient concentrations that are context-appropriate in low-resource settings may significantly and positively shape the nutritional and cognitive status of children, ultimately reducing nutritional and health disparities in the country.

There are multiple strengths to this study. First, the study is robustly designed and includes children from low sociodemographic backgrounds from Guatemala, a country with widespread nutritional deficiencies. Findings from this study suggest an effective scalable opportunity to improve nutritional and developmental health in contexts with widespread poverty. Second, the study highlighted a model that may be easily replicated and adapted to similar contexts in low- and middle-income countries, using community-engaged methods and including key stakeholders such as local governments, nonprofit organizations, and teachers and cooks at the schools.

There are a few limitations to the present study. First, we initially designed the study to include only children in the control group who did not attend any early childcare institution. During the first year of the study, we realized that most children in the control group had attended many types of early childcare institutions. Thus, we had to modify our study design, and we did not ascertain the pedagogical approaches in early childcare institutions from the control group that may have affected the diverse outcomes. Another limitation that may account for the observed differences is the household structure, which was not explored in the study. Family composition may be associated with some of the outcomes explored in this study [46,47]. Additionally, extramural factors, including exposure to violence in high-crime areas in both children from the GCMN and controls, may have also negatively impacted the study outcomes. Lastly, the sample of this study was primarily urban, from the capital city of Guatemala; thus, external validity may be limited to urban, large cities in Guatemala and Central America.

In conclusion, children attending the GCMN are associated with better math and fluid intelligence scores than peers who did not attend these early childhood centers in second grade. Based on these findings, we suggest that the GCMN may offer an affordable, high-quality, comprehensive, early childhood education to children from low-resource settings in Guatemala City in addition to nutrition and micronutrient supplementation assistance. This type of integrated nutrition and high-quality early childhood education approach may be easily replicable in similar contexts and be key to reducing long-term human and economic disparities. Early life experiences are key to an optimal development and help shape the child’s brain, especially during the first years of life. Early childcare institutions are an overlooked, cost-effective opportunity to provide a safe, stimulating environment that could be rich in exploratory opportunities and social play for children, especially to those in poverty conditions and/or suffering from neglect [48]. Additionally, early childhood centers can be a platform for the delivery of health and nutrition services that are even more needed in a country where 61.8% of the population lives in multidimensional poverty and 23.4% in extreme poverty [49].

## Figures and Tables

**Table 1:** Sociodemographic and anthropometric characteristics and test scores of children that received early childhood intervention (“graduates”) vs. age, sex, and location-matched controls ^1^.

	Graduates	Controls	*p*-Value ^3^
Age, years	8.69 (0.39)	8.63 (0.43)	0.139
Female, %	57.0	57.0	1.00
Maternal education, secondary or more	65.1	57.2	0.065
Household Asset Score	5.91 (2.43)	5.54 (2.43)	0.086
Household Income, %			
<2000 GTQ	26.3	28.7	0.281
2000–3000 GTQ	25.7	28.7	
3000–5000 GTQ	24.0	26.9	
>5000 GTQ	24.0	15.6	
Overcrowding, % ^2^	33.7	35.5	0.671
Did not attend an early education institution, %	0	8.8	<0.001

^1^ Values are the means (standard deviations) unless otherwise specified. ^2^ Overcrowding defined as more than three people per habitable room. Ref: Principles and recommendations for the population and housing censuses (revision 2). New York: United Nations; 2007. ^3^ Unadjusted student *t*- or chi-square tests.

**Table 2:** Anthropometry: adjusted linear mixed models and generalized linear mixed models. Parameter estimates and adjusted odds ratios (AOR) shown for children that attended the Guatemala City Municipal Nurseries.

Outcome	Estimate	95% CI	*p*-Value
Height-for-age z-score.	0.14	−0.05–0.33	0.159
BMI-for-age z-score	0.07	−0.17–0.30	0.591
	**AOR**	**95% CI**	***p*-Value**
Stunted (height for age z-score < −2)	0.39	0.07–2.05	0.265
Overweight (BMI-for-age z-score > 2)	1.20	0.65–2.19	0.566

**Table 3:** Estimates of the fixed effects for the cognitive tests and academic average. Estimates shown for children that attended the Guatemala City Municipal Nurseries.

Outcome	Estimate	95% CI	*p*-Value
Mathematics	6.48	2.35–10.61	0.002
Fluid intelligence (Raven’s progressive matrices)	1.20	0.12–2.29	0.029
Receptive vocabulary (Peabody Vocabulary Test)	1.59	−0.31–3.49	0.100
General average	1.07	−0.59–2.73	0.206

## Data Availability

The data presented in this study are available on request from the corresponding author.
